# Computer vision syndrome and associated factors in university students and teachers in Nampula, Mozambique

**DOI:** 10.1186/s12886-023-03253-0

**Published:** 2023-12-13

**Authors:** Dulnério Barbosa Sengo, Abel da Deolinda Bernardo Pica, Isaura Ilorena d’Alva Brito Dos Santos, Laura Mavota Mate, Avelino Nelson Mazuze, Pablo Caballero, Inmaculada López-Izquierdo

**Affiliations:** 1 Instituto Superior de Ciências da Saúde, Av. Tomás Nduda, nr. 977 RC, Cidade de Maputo, Mozambique; 2 Universidade Lúrio, faculdade Ciências de Saúde, Bairro de Marrere, R. nr, 4250 Nampula, Mozambique; 3 Ministério dos Combatentes, Av Mártires Machava, nr. 307, Cidade de Maputo, Moçambique; 4 Universitat d’Alacant, Departamento de Enfermería Comunitaria, Medicina Preventiva y Salud Pública e Historia de la Ciencia, Carretera Sant Vicent del Raspeig s/n, 03690, Sant Vicent del Raspeig, Alacant, Spain; 5 Universidad de Sevilla, Departamento de Física de la Materia Condensada, Av. Reina Mercedes s/n, 41012 Sevilla, Spain

**Keywords:** Asthenopia, Eye fatigue, Visual fatigue, Computers, Handheld, Ergonomics, Universities, Students, COVID-19, Mozambique

## Abstract

**Background:**

Computer Vision Syndrome (CVS) is a complex of eye and visual problems that arise while using a computer or other Video Display Terminal (DVT). With the advent of the COVID-19 pandemic, the use of these DVTs has become indispensable in the lives of students and teachers. This study aims to identify the prevalence of CVS and associated factors in students and teachers at Lúrio University, in Nampula, during the pandemic period.

**Methods:**

This is a cross-sectional study, carried out between November 2020 and March 2021. The validated CVS questionnaire (CVS-Q) and another semi-structured questionnaire on ergonomic risk factors were applied. Odds ratios (OR) and adjusted odds ratios (aOR) were calculated to measure the association between CVS and computer use conditions.

**Results:**

The prevalence of CVS was 76.6%, and the female gender, age ≤ 20 years, levels I, II, III of course, lack of knowledge about ergonomics, use the computer to study, use more than 6 hours daily, absence of anti-reflex treatment, use of other devices and sitting in an inappropriate chair were risk factors for the occurrence of CVS, while being a teacher was a protective factor.

**Conclusion:**

The prevalence of CVS found in this study was high, due to several factors, especially not using ergonomic principles when using computers and other DVTs. There is a need to adopt intervention strategies focused on the most vulnerable groups such as women, age group ≤20 years and students, especially at the first year level, right after entering the University.

## Introduction

The computer is part of a group of devices called the “Video Display Terminal” (VDT), which also includes tablets, e-book readers, smartphones and other digital devices that have emerged with the advancement of technology and increasing socioeconomic development, and have become indispensable in people’s lives, being used for many purposes and in various scenarios such as offices, academic institutions, homes and others [1, 2].

These devices, of course, have benefited society and made life easier for many people, but they can cause damage if used incorrectly. Prolonged use of computers and other VDTs often causes a cluster of symptoms known as Computer Vision Syndrome (CVS) [3–5]. CVS, also known as Digital Eye Strain (DES), is a complex of eye and vision problems that arise while using a computer or other VDTs in near vision activities. They are usually associated with prolonged and uninterrupted focusing of the eyes on the VDT, causing eye muscle tension [6].

Symptoms of CVS include: dry and irritated eyes, eye strain or fatigue, blurred vision, red eyes, burning eyes, excessive tearing, double vision, headache, sensitivity to light, glare, slow focus changes, and changes in colors perception [3, 5].

Many studies have reported as risk factors for CVS occurrence inadequate distance of use, poor lighting in the workplace, glare and/or screen glare, vision problems, the use of contact lenses and inadequate workstation setup [7–9], as well as hours of use and poor posture during use [2, 10].

The CVS is the leading occupational hazard of the twenty-first century and a growing public health concern that significantly contributes to reduced quality of life, decreased efficiency and productivity in the workplace, higher error rates, lower job satisfaction and visual ability compromised [11, 12].

Even before the COVID-19 pandemic, studies reported a prevalence of CVS in computer users between approximately 60 and 80% [3, 5, 6, 10, 13–15]. Worldwide, around 60 million people suffered from CVS and 1 million new cases were detected every year [3, 5, 11, 12].

However, with the COVID-19 pandemic and consequent global lockdown, sudden closure of educational institutions and interruption of teaching and face-to-face learning, digital technologies have gained prominence and the computer has become indispensable for the education and entertainment of any child and student, and with that, the hours of VDTs use increase considerably [7, 8, 16].

During the COVID-19 pandemic, many institutions adopted the blended teaching modality as an obligation, and after the pandemic some continued to adopt it as an option, as is the case of Lúrio University (LU).

Given the low availability and use of personal protective equipment, high workload, limited break time and little knowledge of ergonomics during computer use in developing countries the burden of CVS is very high [3, 12].

In Mozambique, to date, no published studies have been found that report the prevalence and risk factors of CVS. Therefore, this study aims to determine the prevalence of CVS and associated factors in students and teachers at LU.

## Material and methods

This is a cross-sectional study carried out at LU, at Faculty of Health Sciences (FHS) and Faculty of Architecture and Physical Planning (FAPP), on the university campus of Marrere, Nampula. LU is a public university, located in northern Mozambique, with its three hubs located in the three provinces of northern Mozambique, namely: Nampula, Niassa and Cabo Delgado. The Nampula hub is composed by the FHS, FAPP and UniLúrio Business School (UBS). The Marrere Campus has two organic units (FHS and FAPP).

The FHS contains 8 courses, namely: degree in general medicine, degree in dental medicine, degree in pharmacy, degree in nutrition, degree in optometry, degree in nursing, degree in health administration and management, and clinical psychology. On the other hand, FAPP has only 2 courses: a degree in architecture and physical planning, and a degree in urbanism and spatial planning.

### Ethical aspects

This study was previously approved by the Institutional Committee on Bioethics for Health of Lúrio University (CIBSUL) with ref.: 26/Out/CBISUL/20, on October 29, 2020. All interviewees were previously informed about the nature of the study and participated in the study by signing an informed consent form.

### Sampling and population

The study population comprised a total of 2097 individuals among students (1864; 88.8%) and teachers (233; 11.1%) of FHS and FAPP. However, for an estimate of unknown proportion, since no previous studies were found in Mozambique, with a confidence level of 95% and an accuracy of 5%, a minimum sample size of 325 participants was considered [17], and randomly selected. The sample was proportionally stratified by occupation: 289 students (88.8%) and 36 teachers (11.1%).

### Participation criteria

#### Inclusion criteria

Students and teachers who use a computer at least 3 hours a day and who signed the informed consent form were included in the study.

#### Exclusion criteria

Students and teachers with evident ocular deviations, infectious keratoconjunctivitis, allergic keratoconjunctivitis, blepharitis, ocular trauma, previous eye surgery or who were undergoing topical treatment were excluded.

### Data collection

Data were collected between November 2, 2020 and March 31, 2021. During this period, the teaching modality adopted by LU in view of the COVID situation was the hybrid model (with in-person and online classes). Therefore, data collection was carried out in person, during the weeks of face-to-face classes.

### Instruments and data collection process

The collection of symptoms and diagnosis of CVS was based on the computer vision syndrome questionnaire (CVS-Q) developed and validated by Seguí M, et al. [18].

This questionnaire first underwent a translation and cultural adaptation into Portuguese in the Mozambican context following the criteria defined by the American Association of Orthopedic Surgeons [19]: translation, synthesis of translations, back translation, committee of experts and pre-test.

Although the psychometric properties of the adapted version into Portuguese were not evaluated, its original version showed sensitivity and specificity values of above 70%, with good test-retest repeatability, for the score by the Intraclass Correlation Coefficient (ICC = 0.802; 95% CI, 0.673–0.884) and for the diagnosis of CVS by Cohen’s kappa (k = 0.612; 95% CI, 0.384–0.839). The area under the ROC curve was 0.826 (*p* < 0.001). Therefore, this questionnaire has good psychometric properties al [18].

This is a self-administered questionnaire with 16 items to assess the symptoms perceived by the interviewee, their frequency (categorised into: never, occasionally and often or always) and intensity (categorised into: moderate and severe), and values are assigned to each category as follows: Never = 0 value, Occasionally = 1 value, Frequently or always = 2 values, Moderate = 1 value and Severe = 2 values. For each symptom, the frequency value is multiplied by the intensity value and the result is coded with the following score: 0 value = 0 points, 1 value or 2 values = 1 point and 4 values = 2 points. At the end, the points for all the items are added together and a score of CVS-Q ≧ 6 is considered to have CVS.

A rapid review was performed in Pubmed and google scholar, using the following search equation: (“asthenopia” OR “computer visual syndrome” OR “visual fatigue”) AND (“computer terminals” OR “video display terminal” OR “workplace”) AND (“work conditions” OR “risk factors”), to identify the risk factors for the occurrence of symptoms during computer use commonly considered in the literature, having identified the following variables and factors: occupation, gender, age, knowledge about ergonomics and CVS, eye health state, computer and other VDTs usage time, usage purpose, usage distance, anti-glare treatment on screens, screen brightness adjustment, screen height, usage breaks, activities during breaks, workplace lighting, posture [2, 4, 20–24].

Subsequently, a semi-structured questionnaire on ergonomic risk factors for computer use was prepared, with an illustration (showing the ideal posture for computer use) attached to the questionnaire to better guide the interviewee with respect to issues associated with posture.

Both questionnaires (CVS-Q and questionnaire on ergonomic risk factors of computer use) were pre-tested in an exploratory way (interview) in 48 participants randomly chosen, among professors and students computer users. All questions in which at least 15% of the participants had difficulties to understand were identified and adjusted, as already done in previous studies [25].

The CVS-Q questionnaire was self-administered, while the ergonomic risk factors questionnaire, by its nature, was administered by two interviewers (D.B.S. and A.D.B.P.).

### Definition of variables

Socio-demographic data were collected (age, gender, occupation, course, course level), eye condition (eye symptoms and their frequency and intensity, and use of contact lenses or glasses), knowledge about ergonomics (ergonomics principle, CVS, rule 20–20-20), computer usage habits (purposes of use, hours of use, breaks, distance, anti-glare monitor treatment, monitor brightness adjustment, use of other VDTs), working environment (lighting, light source and use of air conditioning) and posture during use (sitting on the bed or mat, sitting on the simple chair, lying down or sitting on the appropriate ergonomic chair, monitor height, postural breaks, postural symptoms).

### Data analysis

Descriptive data were organized into tables and graphs. To study the association between the dependent variable (CVS) and independent variables (socio-demographics, use of glasses or contact lenses, knowledge about ergonomics, computer use habits, work environment and posture during computer use), the odds ratio (OR) and adjusted odds ratio (aOR) were calculated, with a confidence interval of 95%. All data analyses were performed using Statistical Package for the Social Science version 23.0 (SPSS Inc., Chicago, IL, USA).

## Results

### Description of socio-demographic characteristics and association with the presence of CVS

This study had a sample of 325 participants, aged between 18 and 52 years (with a mean age of 23.2 years, S.D. 5.9). Most participants were male (68.9%) and students (88.9%) (Table 1). The prevalence of CVS among participants was 76.6% (95% CI: 71.7–81.2).

**Table 1:** Description of socio-demographic characteristics and association with the presence of CVS

Variables	Sample	With CVS		
	N (%)	N (%)	OR (95%CI)	aOR (95% CI)
Gender				
Male	224 (68.9)	158 (70.5)	1	1
sFemale	101 (31.1)	91 (90.1)	3.8 (1.9; 7.8)	5.1 (1.8; 14.2)
Age (years)				
≤ 20^a^	149 (45.8)	124 (83.2)	2.0 (1.2; 3.5)	1
21–30^a^	148 (45.5)	108 (73.0)	1.5 (0.9; 2.4)	n.s
31–40^a^	22 (6.8)	14 (63.6)	2.0 (0.8; 4.9)	n.s
> 40^a^	6 (1.8)	3 (50.0)	3.4 (0.7; 17.1)	n.s
Occupation				
Student	289 (88.9)	230 (79.6)	1	1
Teacher	36 (11.1)	19 (52.8)	0.3 (0.1; 0.6)	0.1 (0.0; 0.3)
Course				
Optometry	17 (5.2)	12 (70.6)	1	1
Pharmacy	29 (8.9)	22 (75.9)	1.3 (0.3; 5.0)	n.s
General Medicine	26 (8.0)	20 (76.9)	1.4 (0.3; 5.5)	n.s
Dental medicine	31 (9.5)	24 (77.4)	1.4 (0.4; 5.5)	n.s
Nutrition	33 (10.2)	26 (78.8)	1.5 (0.4; 5.9)	n.s
Nursing	28 (8.6)	20 (71.4)	1.0 (0.3; 3.9)	n.s
Architecture	49 (15.1)	44 (89.8)	3.7 (0.9; 14.8)	n.s
Urbanism	52 (16.0)	38 (73.1)	1.1 (0.3; 3.8)	n.s
Administration	34 (10.5)	24 (70.6)	1.0 (0.3; 3.6)	n.s
Psychology	26 (8.0)	19 (73.1)	1.1 (0.3; 4.4)	n.s
Course level				
II	88 (27.1)	80 (90.9)	12.5 (3.8; 40.7)	10.9 (2.3; 50.5)
III	99 (30.5)	74 (74.7)	3.7 (1.3; 10.4)	1.9 (0.5; 7.8)
IV	65 (20.0)	48 (73.8)	3.5 (1.2; 10.4)	1.8 (0.4; 8.1)
V	55 (16.9)	39 (70.9)	3.0 (1.1; 9.1)	1.6 (0.4; 7.3)
VI	18 (5.5)	8 (44.4)	1	1
Total	325	249 (76.6)		

^a^ The reference group is the rest of the population; *CI* confidence interval, *OR* odds ratio, *aOR* adjusted odds ratio

The ORs show a statistical association with the female gender (OR:3.8), age group ≤20 years (OR:2.0) and course levels II, III, IV and V (with OR:12.5, OR:3.7, OR: 3.5 and OR:3.0, respectively) as risk factors for the occurrence of CVS, while being a teacher was a protective factor (OR:0.3) for the occurrence of CVS.

However, in the multivariate model, aOR shows association only with female gender (aOR: 5.1) and course level II (aOR: 10.9) as risk factors, while being a teacher was protective (aOR: 0.1) (Table 1).

### Conditions of computer use and association with the presence of CVS

Among the interviewees, the majority (72.6%) did not present any type of correction (either through eyeglass or contact lenses), and had no knowledge of ergonomics, ergonomic principles of computer use, the 20–20-20 rule and CVS.

Most of the participants had no knowledge about ergonomics, ergonomic principles of computer use, the 20–20-20 rule and CVS, and have not adopted good computer use practices, that is, they do not take breaks during use (48.0%), others during the break do activities that need near vision (37.5%), use the computer less than 40 cm (81.5%), their monitors do not have anti-glare treatment (58.2%) and in addition to a computer, they have used smartphones (58.5%). Regarding posture during computer use, most have been sitting in inappropriate chairs (57.8%), the top of the monitor is not slightly below eye level (85.2%) and have not taken postural breaks during computer use (70.5%).

Most participants have used the computer in a workplace with good lighting (42.8%) and without air conditioning (72.6%). The predominant type of lighting was incandescent (42.2%) (Table 2).

**Table 2:** Conditions for computer use and association with CVS

Conditions	Sample	With CVS		
	N (%)	N (%)	OR (95%CI)	aOR (95% CI)
Eyeglass wearer				
No	236 (72.6)	181 (76.7)	1.0 (0.6; 1.8)	n.s
Yes	89 (27.4)	68 (76.4)	1	1
**Knowledge**				
About ergonomics				
No	276 (84.9)	214 (77.5)	1.4 (0.7; 2.7)	n.s
Yes	49 (15.1)	35 (71.4)	1	1
About ergonomic principles				
No	289 (88.9)	227 (78.5)	2.3 (1.1; 4.8)	4.5 (1.2; 16.2)
Yes	36 (11.1)	22 (61.1)	1	1
About 20–20-20 rule				
No	270 (83.1)	220 (81.5)	3.9 (2.1; 7.3)	5.1 (2.1; 12.5)
Yes	55 (16.9)	29 (52.7)	1	1
Abaout CVS				
No	246 (75.7)	190 (77.2)	1.1 (0.6; 2.1)	n.s
Yes	79 (24.3)	59 (74.7)	1	1
**Habits during computer use**				
Computer use purposes				
Work	40 (12.3)	29 (72.5)	1	1
Study	90 (27.7)	82 (91.1)	3.8 (1.4; 10.6)	4.8 (1.1; 20.8)
Study and leisure	168 (51.7)	119 (70.8)	0.9 (0.4; 1.9)	n.s
Study, work and leisure	27 (8.3)	19 (70.4)	0.9 (0.3; 2.6)	n.s
Hours of computer use per day				
</= 6 h	173 (53.2)	115 (66.5)	1	1
>6 h	152 (46.8)	134 (88.2)	3.8 (2.1; 6.7)	7.7 (3.1; 19.1)
Activities during breaks				
Without breaks	156 (48.0)	118 (75.6)	1.3 (0.6; 2.7)	n.s
activities that require near vision	122 (37.5)	98 (80.3)	1.7 (0.8; 3.7)	n.s
activities that do not require near vision	47 (14.5)	33 (70.2)	1	1
Computer use distance				
≥40 cm	60 (18.5)	45 (75.0)	1	1
<40 cm	265 (81.5)	204 (77.0)	1.1 (0.5; 2.1)	n.s
Monitor with anti-glare				
No	189 (58.2)	161 (85.2)	3.1 (1.8; 5.3)	2.5 (1.2; 5.6)
Yes	136 (41.8)	88 (64.7)	1	1
Have you adjusted the monitor brightness?				
No	134 (41.2)	103 (76.9)	1.0 (0.6; 1.7)	n.s
Yes	191 (58.8)	146 (76.4)	1	1
Have used other VDTs				
None	55 (16.9)	23 (41.8)	1	1
Smartphone	190 (58.5)	156 (82.1)	6.3 (3.3; 12.2)	13.8 (5.4; 35.6)
Smartphone and tablet	80 (24.6)	70 (87.5)	9.7 (4.1; 22.8)	29.5 (8.7; 100.1)
**Workplace**				
Lighting				
Good	139 (42.8)	102 (73.4)	1	1
Poor	100 (30.8)	80 (80.0)	1.5 (0.8; 2.7)	n.s
Dark	86 (26.5)	67 (77.9)	1.3 (0.7; 2.4)	n.s
Light source				
Natural light	65 (20.0)	49 (75.4)	1	1
Incandescent	137 (42.2)	108 (78.8)	1.2 (0.6; 2.4)	n.s
Fluorescent	123 (37.8)	92 (74.8)	1.0 (0.5; 1.9)	n.s
Air conditioning in the workplace				
Without	236 (72.6)	183 (77.5)	1	1
With	89 (27.4)	66 (74.2)	0.8 (0.4; 1.5)	n.s
**Posture**				
Computer use position				
Sitting (bed/mat/floor)	70 (21.5)	57 (81.4)	1.8 (0.7; 5.0)	n.s
Sitting (inappropriate chair)	188 (57.8)	145 (77.1)	1.4 (0.6; 3.4)	4.8 (1.3; 18.4)
Lying down	37 (11.4)	26 (70.3)	1.0 (0.3; 2.9)	n.s
sitting (appropriate ergonomic chair)	30 (9.2)	21 (70.0)	1	1
Top of the monitor slightly below eye level				
No	277 (85.2)	214 (77.3)	1.2 (0.6; 2.5)	n.s
Yes	48 (14.8)	35 (72.9)	1	1
Takes postural breaks every 1 hour at least				
No	229 (70.5)	181 (79.0)	1.5 (0.9; 2.7)	n.s
Yes	96 (29.5)	68 (70.8)	1	1
Have you had back, spine, shoulder or neck pain				
No	129 (39.7)	96 (74.4)	1	1
Yes	196 (60.3)	153 (78.1)	1.2 (0.7; 2.1)	n.s

The ORs show a statistical association between CVS and lack of knowledge about ergonomic principles of computer use (OR: 2.3) and the 20–20-20 rule (OR: 3.9), as risk factors for the occurrence of CVS.

Regarding computer usage habits, CVS had a statistical association with computer use for study purposes (OR: 3.8), use more than 6 hours a day (OR: 3.8), monitor without anti-reflective treatment (OR: 3.1), use of other VDTs (smartphone or smartphone and tablet, OR: 6.3, OR: 9.7, respectively) and sitting in inappropriate chairs (aOR: 4.8) as risk factors for the occurrence of CVS (Table 2).

### Symptoms reported while using the computer

**Fig. 1:**
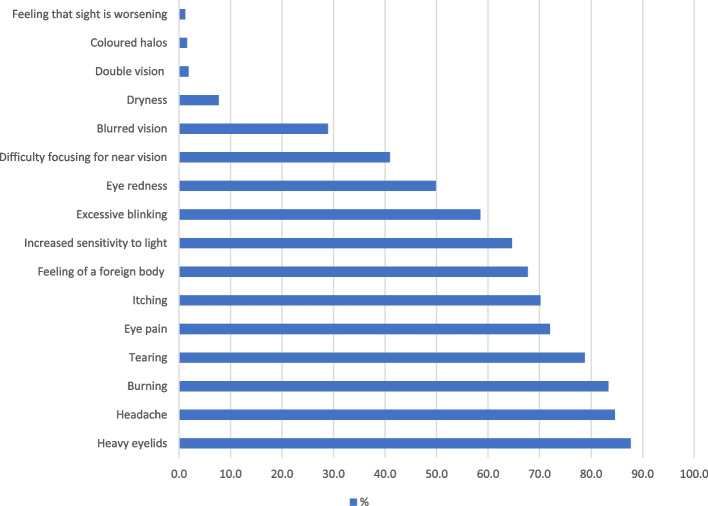
Proportion of eye symptoms reported by respondents

Among the symptoms reported during computer use, Heavy eyelids was the most frequent (87.7%), followed by headache (84.6%), burning (83.4%), tearing (78.8%) and eye pain (72.0%). The least cited symptoms were Feeling that sight is worsening (1.2%), Coloured halos (1.5%) and double vision (1.8%) (Fig. 1).

## Discussion

COVID-19 was declared a pandemic by the World Health Organization (WHO) on March 11, 2020 and is rapidly spreading across the world. As a result, students and teachers at all levels needed to quickly adapt to educational changes arising from the circumstances of the pandemic [7, 26].

Overnight, digital technologies gained prominence in the educational field, as distance learning gained more space, as the only viable teaching modality at the time [7]. However, this paradigm shift resulted in greater exposure to the VDTs.

In Mozambique, to date, no published studies on the prevalence of CVS in students or teachers have been found. Therefore, this study aims to identify the prevalence of CVS and associated factors in students and professors at LU during the COVID-19 pandemic.

### Prevalence of CVS

In this study, the prevalence of CVS found was 76.6%, which is higher than the prevalence found in studies carried out with university students in Peru [27] and Iran [28], which obtained a prevalence of 62.3 and 48.7%, respectively, while in Thailand [29] and Paraguay [30] the prevalence found was higher (81.0 and 82.5%, respectively).

The studies carried out in Peru [27] and Iran [28] took place before and at the beginning of the pandemic, respectively, and in these periods, supposedly, exposure to DVTs was lower, so the prevalence of CVS was lower in these studies. Another aspect to take into account is the exclusion criteria, for example in the Iranian study, the exclusion of participants with uncorrected refractive errors or any other ocular condition that could contribute to the occurrence of asthenopia may have led to a lower prevalence of CVS in this study.

The studies carried out in Thailand [29] and Paraguay [30], as well as the present study, were carried out during the COVID-19 pandemic, so it is understandable that they found a higher prevalence of CVS, however, in the present study, participants with evident ocular deviations, some ocular pathology or undergoing topical treatment were excluded, which may have contributed to the lower prevalence among the three studies, since these can contribute to the appearance of symptoms associated with CVS.

### Factors associated with the occurrence of CVS

#### Gender

In this study, the female gender was identified as a risk (OR:3.8) for the occurrence of CVS, corroborating the results found in the study carried out in Thailand (OR:2.45) [29], which may be due to the fact that evaporation of the tear film in women is greater [31], because of the difference in hormone levels in men and women (including androgen and estrogen) and female hormonal cycles (menstruation, pregnancy, menopause) [32] leading to symptoms of dry eye, which in turn are similar to those of CVS.

#### Age

The prevalence of CVS decreases with increasing age, and the age group ≤20 years was presented as a risk (OR: 2.0) for the occurrence of CVS. These results are in line with those found in a study carried out in Thailand [29], in which increasing age was a protective factor for the occurrence of CVS (OR: 0.86), which may be due to the fact that the younger ones, because they are attending the first years of college, have more theoretical subjects and consequently more online classes.

#### Course level

The prevalence of CVS tended to decrease with increasing level in the course, and levels II, III, IV and V were presented as risk factors for the occurrence of CVS, which may be associated with the load of theoretical classes in the first years of college, which has a tendency to decrease as practices increase. Therefore, in the first years of college, there are more online classes, greater exposure to DVTs and, therefore, a greater risk of occurrence of CVS.

#### Occupation

The prevalence of CVS is lower in teachers, and being a teacher was a protective factor for the occurrence of CVS in relation to students, which may be associated with the volume of online work taking into account the number of subjects in which each category is involved. Students take an average of 6 subjects per semester, while professors teach an average of 3 subjects, which can result in fewer hours of exposure than students.

Social media, educational tasks and long hours of study at the undergraduate level result in increased near vision activities and eye exposure to smartphones, computers and tablets, making students more vulnerable to CVS [33].

#### Knowledge of ergonomic principles of computer use and 20–20-20 rule

The lack of knowledge about ergonomic principles (OR: 2.3) and the 20–20-20 rule (OR:3.9) were identified as a risk factor for the occurrence of CVS, in line with the results found in the study carried out in Ethiopia [3], where good knowledge on safe use of computer and prevention mechanisms of adverse effect of computer were protective (OR:0.33) for the occurrence of CVS. Therefore, having knowledge about ergonomic principles can contribute to the adoption of good habits of computer use, thus avoiding the occurrence of symptoms. However, in a study carried out in Saudi Arabia [4], being aware of the 20–20-20 rule was a risk factor (OR:1.71) for the occurrence of CVS, which was associated with a possible association between knowledge of ergonomics and greater daily use of the computer, that is, those who use computers the most have more knowledge about ergonomics, but excessive use is a risk factor for the occurrence of CVS.

Therefore, it is not enough to have knowledge about ergonomics, the application of this knowledge will be decisive for the non-occurrence of CVS.

#### Computer use purposes

Computer use just for studying was a risk (OR:3.8) for the occurrence of CVS with respect to use only for work (reference category). Computer use for work is associated with the occupation “teacher”, and being a “teacher” was a protective factor for the occurrence of CVS. On the other hand, “studying” is an activity that demands a lot of visual effort and concentration, so the propensity for ocular symptoms to occur is also greater. The teacher’s job is to teach, which is an act that allows more changes of focus between near and distant vision, and leisure activities, in general, do not require permanent decoding of characters (reading), so they require less effort.

#### Hours of computer use per day

The prevalence of CVS is higher in the group that uses a computer more than 6 hours a day. However, using the computer more than 6 hours a day was a risk factor for the occurrence of CVS (OR:3.8), corroborating the findings in the study carried out in Ethiopia, Saudi Arabia and Malaysia [3, 4, 34], in which longer computer use (> 4.6 h, > 5 h and > 5 h, respectively) constituted a risk for the occurrence of CVS.

#### Monitor without anti-glare treatment

Glare is a visual sensation resulting from the imbalance of light between the computer screen and its surroundings [35], with light reflections on the screen, excess light in the field of vision and reduced contrast, which can fade the images of characters on the screen [36]. Therefore, anti-glare treatment is a layer of coating on the screen that diffuses light instead of reflecting it, thus preventing it from focusing on the user’s eyes [22].

The use of a monitor without anti-glare treatment was a risk factor (OR: 3.1) for the occurrence of CVS. These results coincide with the results of studies in Thailand (OR: 2.24) and Sri Lanka (OR: 1.02), in which the absence of anti-glare treatment on the monitor also represented a risk for the occurrence of CVS [5, 29]. However, a different result was found in another study in Ethiopia [24] in which the use of anti-glare treatment did not reduce the risk of CVS symptoms. However, glare from the window or ceiling light can cause reflections on surfaces and the computer screen (overlapping the images), which can alter the brightness of the visual field and reduce contrast, causing visual symptoms. It is believed that potentially conflicting brightness on a computer monitor may induce inappropriate accommodation responses [22] and contraction of the orbicularis oculi (affecting blink rates) resulting in visual symptoms) [29].

#### Use of other VDTs devices

The use of other VDTs, in addition to the computer, presupposes more hours of exposure to VDTs, which in turn results in a higher risk of CVS according to the results of this study. Therefore, those who used, in addition to a computer, a smartphone and a tablet had a higher risk of developing CVS.

#### Posture while using computer

The use of an inappropriate chair (non-ergonomic) was presented as a risk factor (aOR:4.8) for the occurrence of CVS. The use of an ergonomically appropriate chair favors the adoption of an adequate posture when using computer, which presupposes a lower risk for the occurrence of symptoms according to the study carried out in Ethiopia [24], in which an inappropriate position while using a computer was a risk (OR: 2.56) for CVS occurrence.

However, we must remember that human eyes need to adjust to see objects from different distances, such as changing the size of the pupil, lengthening or shortening the lens (crystalline) to change the focus of the eye, and contracting the extraocular muscles to converge the two eyes [5], and to focus an image closely, the ciliary muscle needs to contract to relax the zonules [37].

Therefore, excessive or prolonged computer use, as well as other VDTs, as it usually occurs in activities performed in near vision, where the user needs to maintain accommodation (contracted ciliary muscle) and convergence (contracted extraocular muscles) to maintain the sharp image, results in symptoms of fatigue and spasms of the involved muscles.

The human visual system is not intended for focusing on characters generated electronically by VDTs. It responds perfectly to images that have sharp edges and good background contrast (eg solid black letters on a white background). Each pixel is brightest in the center, with the brightness decreasing toward the outer edges. The human eyes find it very difficult to focus on the pixel characters [5, 38].

Another factor that contributes to the occurrence of CVS is the blue light emitted by VDTs, as studies have shown that reduced exposure to blue light reduces the occurrence of visual fatigue [35].

Therefore, this study brings to light data about a problem that many suffer and few know is CVS. There are no published data in Mozambique on the prevalence and risk factors of CVS, which makes it difficult to perceive the magnitude of the problem and limits the ability to intervene on the problem that plagues workers, students, teachers and other computer users, interfering in their productivity and quality of life. These data allow the proposition of more focused intervention strategies taking into account the characteristics of the most vulnerable. Therefore, intervention research that seeks to educate and raise awareness about the ergonomic principles of computer use and other VDTs is necessary to change these indicators, as it has become evident through this study that a lack of knowledge about the ergonomic principles of computer use can be a risk factor for the occurrence of CVS. At the LU level, the inclusion of a module or thematic unit of ergonomics in the subject of “Introduction to Informatics” that is taught in all courses during the first year, would be a starting point to spread knowledge and make students aware of good habits of VDTs use.

The results of this study lead us to reflect on the benefits and harms of using VDTs. Undoubtedly, the use of VDTs is irrevocable nowadays, data has shown that dependence on these equipments is increasing, due to a diversity of needs and contexts. At LU, for example, the hybrid teaching modality is here to stay, and VTDs are important tools in the teaching process. However, the way to use these devices can really be a differentiator for the occurrence or not of CVS.

### Recommendations

CVS prevention requires a multidisciplinary intervention, and taking into account the risk factors identified in this study, it would be important to consider the following recommendations: regulate the hours of computer use and other digital devices, using them intermittently, and increase the intervals at rest looking at distant objects to allow the ciliary muscle to relax and relieve its tension. Adopting the 20–20-20 rule, that is, every 20 minutes, looking at something 20 ft (6 m) away for 20 seconds, is one of the most recommended mechanisms [36, 39]. Adequate lighting in the work environment is essential to prevent CVS, since when this is deficient the user needs to bring the object too close, compromising their posture and increasing accommodation (contraction of the ciliary muscle) and convergence (contraction of the extraocular muscles) [36]. It is therefore essential to ensure good lighting (between 500 and 1000 lx for visually demanding activities) [21], evenly distributed and positioned so that it does not focus on the eyes or screen (avoid standing behind the user). Use a computer with a screen that has an anti-glare filter and glasses with a blue light filter and anti-glare filter to increase contrast, as well as lubricating the eyes with eye drops when using the computer [36, 39]. Another indispensable aspect is proper posture when using the computer (neutral posture of the neck and spine without flexion, inclination and/or rotation), which is why it is important to use an appropriate ergonomic chair [21]. The distance when using digital devices should be at least 40 cm [22].

### Limitations

The main limitation of this study is the fact that eye examinations were not performed to know the real eye status of the participants, since some eye conditions such as uncorrected refractive errors, accommodative and binocular vision anomalies, and dry eye increase the risk of CVS. Psychological factors such as daily stress and a weakened mental state can also influence the occurrence of CVS [33], as well as some environmental and ergonomic factors such as the positioning of lights and windows, temperature and relative humidity, some postural aspects (such as neck and spine posture), eye-screen angle, gaze angle and angle of inclination of the screen in relation to the horizontal [21, 22]. Therefore, these risk factors were not studied in this study.

On the other hand, during COVID-19, the Portuguese CVS-Q version used was not yet valid, however, it is assumed that when this article is published, the validated questionnaire will be available on the questionnaire’s website (https://cvs-q.com/).

## Conclusion

The COVID-19 pandemic has brought a paradigm shift in teaching at a global level. Distance learning and the use of information and communication technologies gained prominence. Since then, Lúrio University has adopted a hybrid teaching model (with in-person and online classes). In this context, exposure to VDTs is higher, for this reason the prevalence of CVS found in this study was high. It was observed that most of the participants are not aware of the ergonomic principles of using computers and other DVTs, therefore, they cannot adopt healthy habits of use of these equipments. Several factors detected in this study (related to hours of use, anti-reflection on the screen, use of other VDTs and posture) can be easily controlled in order to avoid CVS. There is a need to adopt intervention strategies focused on the most vulnerable groups such as women, age group ≤20 years and students, especially at the first year level, right after entering the University.

## Data Availability

The datasets used and analyzed during the current study are available from the corresponding author upon request.

## Abbreviations

VDT: Video Display Terminal
CVS: Computer Vision Syndrome
DES: Digital Eye Strain
LU: Lúrio University
FHS: Faculty of Health Sciences
FAPP: Faculty of Architecture and Physical Planning
UBS: UniLúrio Business School
CIBSUL: Institutional Committee on Bioethics for Health of Lúrio University
CVS-Q: Computer vision syndrome questionnaire
ICC: Intraclass Correlation Coefficient
OR: Odds Ratio
aOR: adjusted odds ratio
